# Perceptions and Needs of Primary Healthcare Providers Regarding Electricity Shortages and Blackouts: A Qualitative Study Using a Realistic Narrative Approach

**DOI:** 10.3389/ijph.2026.1609319

**Published:** 2026-04-20

**Authors:** Enzo Baquet, Paul Tarteret, Agathe Deschamps, Amel Filali, Rose-Anna Foley, Valérie D’Acremont

**Affiliations:** 1 Global and Environmental Health, Centre for Primary Care and Public Health (Unisanté), Lausanne, Switzerland; 2 STEEP Lab, Inria, University Grenoble Alpes, CNRS, Grenoble INP, LJK, Inria Centre de Recherche Grenoble Rhone-Alpes, Montbonnot-Saint-Martin, France; 3 Research and Innovation, School of Health Sciences (HESAV), Lausanne, Switzerland; 4 Faculty of Biology and Medicine, University of Lausanne, Lausanne, Switzerland

**Keywords:** blackout, crisis preparedness, electricity shortage, primary care practices, system resilience

## Abstract

**Objectives:**

To understand how primary healthcare providers perceive a hypothetical electricity supply interruptions (ESI) situation (electricity shortages and blackouts), and their needs for a better preparation and response.

**Methods:**

In Canton of Vaud, Switzerland, interviews with 16 experts of 13 institutions/organisations involved in ESI management were conducted to develop a locally adapted ESI scenario. Perceived risk, knowledge, capacity to adapt, and needs of 8 private practice physicians and nurses were then explored through semi-structured, scenario-based interviews.

**Results:**

Although they considered ESI unlikely and were aware of their heavy dependence on electricity-powered administrative and medical tools, healthcare professionals were willing to continue treating their patients in their own practices or in suitable primary care centres. However, better communication with public health stakeholders, participation in training sessions and to the development of checklists closer to their needs, and a plan indicating them their best possible location would be necessary.

**Conclusion:**

Redefining the role of primary healthcare providers in crises through better training, information, and integration as cooperative partners could represent a key opportunity to enhance the resilience of the whole healthcare system.

## Introduction

Healthcare professionals will increasingly face crisis related to systemic risks [1–3]. These multiple risks, such as climate change, geopolitical tensions, pandemics, and critical resource shortages (water, energy, internet access and telecommunications, medications, personnel) often unfold in a cascade affecting all sectors of society. They expose both healthcare professionals and facilities to complex situations where the demand for care increases while resources are limited [4].

Electricity supply interruptions (ESI), either due to electricity shortages (ES) or blackouts, are more frequent due to imbalance between supply and demand, increase of extreme weather events due to climate change, cyberattacks and wars [5–7]. ES, a situation in which the available generation capacity is insufficient to meet demand, can lead to sustained or cyclical constraints on electricity production, delivery and consumption. Blackouts, such as those that affected Spain and Portugal in April 2025 [8], are defined as large-scale, unplanned, and prolonged losses of electrical power affecting entire regions or countries, causing major disruptions to essential services [9].

Primary care facilities, at the heart of territories and communities, manage most of the population’s health issues. During crises, they thus constitute the first line of defence against hospital overload [10]. Vulnerable populations, such as older people and those with comorbidities, are particularly at risk [4], especially when dependent of electricity-powered treatment such as oxygen. The ability of primary healthcare providers to continue operating despite major disruptions therefore directly determines the resilience of the entire community they serve.

This study aims to explore how primary healthcare providers, private practitioners (PP) and home-visiting nurses (HVN) in Switzerland, perceive a hypothetical ESI situation, using a realistic narrative method to identify their needs for a better response to crisis.

## Methods

### Study Design

Interviews were conducted with experts involved in ESI preparation or management to build a plausible and locally adapted ESI scenario. Semi-structured interviews using this scenario were then conducted with healthcare professionals to explore their perception – used in this paper as a synonym for point of view –, understanding and readiness for such events. The interview guides benefited from the expertise of a health anthropologist (RAF). One or two interviewers at a time conducted the interviews (EB, VDA, AD, AF).

### Settings and Participants

The study took place in the Canton of Vaud, in French-speaking western Switzerland, which had 855′700 inhabitants in 2024. Around 150,000 of them are residents of the capital, Lausanne. The remainder live in rural villages and smaller cities. ESI experts were identified as professionals from various sectors working in institutions or organisations related to electricity or involved in preparation ESI plans. Physicians and nurses from different geographical areas and with varying levels of experience, working in private practices mostly outside major cities, were selected. A snowball sampling method was used to achieve relative variability in sociodemographic criteria, trying to reach the required number of participants to allow data saturation.

### Scenario Development

**FIGURE 1:**
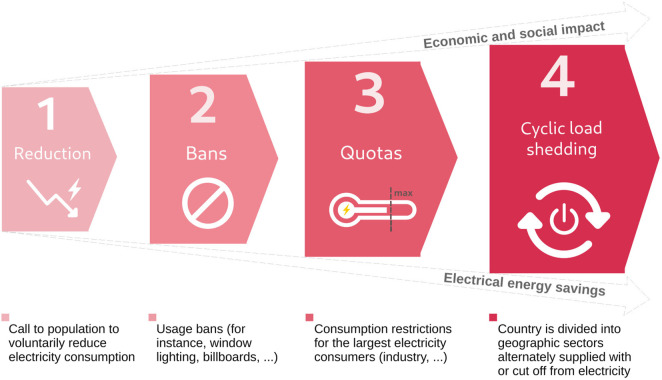
Gradation of anticipated restriction measures in case of electricity shortage in Switzerland (OSTRAL plan) (Perceptions and Needs of Primary Healthcare Providers Regarding Electricity Shortages and Blackouts: A Qualitative Study Using a Realistic Narrative Approach, Switzerland, 2025). (“prohibition” icon by Ardiansyah, from thenounproject.com CC BY 3.0; “circle arrow” icon by Cuputo, from thenounproject.com CC BY 3.0; “Thermometer” icon by Looo, from thenounproject.com CC BY 3.0; “bolt” icon by Jake Schirmer, from thenounproject.com CC BY 3.0).

The fictional narrative scenario of an electricity shortage, followed by a blackout, was developed to help primary healthcare providers contextualize the complex societal impact of electricity interruptions. The cascading events were based on the plan of progressive electricity supply restrictions in four phases, called OSTRAL, developed by Switzerland to manage electricity shortages (Figure 1). This plan does not include a blackout and aims to avoid such outcome at all costs.

The narrative was structured around three fictional characters from different sectors, taking specific actions. Symbols were used to make the scenario easily understandable without constraining participants' perspectives. Participants were asked to react to the five scenes of the scenario corresponding to the four OSTRAL phases, as well as to a situation of blackout. These scenes served as ‘narrative seeds’ to reveal participants' reflections on the repercussions of an ESI on different sectors of the society [11]. We deliberately chose not to include a fictional character working in the healthcare sector, to let participants imagine their own scenario from the perspective of their potential role in such circumstances. Participants were encouraged to continue the narrative and describe their decisions in this situation [12, 13].

### Data Collection

Interviews based on a narrative story completion method were chosen for their ability to gather information beyond direct personal experiences by including discourse and sociocultural representations [12–14]. Informed consent was obtained, and participants were assured of confidentiality, anonymisation of the data, and the option to end the interview at any point. The healthcare providers interviews, using the scenario in a flexible way (interview guides provided in the Supplementary material), were conducted by pairs of male and female researchers from medical, nursing or engineering backgrounds (EB, VDA AD and AF). The research team made sure no prior relationship to the commencement of the study interfered in the process. Verbatim of audio-recorded interviews were transcribed for analysis, followed by an informal debate between the coder and the other interviewer to ensure common understanding. For ESI experts’ interviews (not audio-recorded), detailed summaries were written down based on the researchers’ notes.

### Data Analysis

Thematic analysis following Clarke and Braun’s approach was used to identify key themes in the data [15]. Qualitative analysis of the interviews was performed using QualCoder (Massachusetts Institute of Technology, Cambridge MA, USA). Coding was carried out by one researcher (EB) and discussed with a health anthropologist specialized in qualitative methodologies (RAF). Initial coding was completed by assigning codes in a line-by-line fashion to the data. Codes were then interrogated and collapsed based on similarity to form themes and verbatim quotations were extracted to illustrate major themes [15]. Qualitative data supporting this article are not publicly available due to ethical reason. For any questions, please contact the documentation and data unit at the Center for Primary Care and Public Health, University of Lausanne (Unisanté), through the institutional data repository: https://data.unisante.ch.

## Results

### Information Retrieved From ESI Experts and Scenario Creation

Sixteen interviews were conducted with key ESI professionals from 13 different institutions or organizations: the regional health department, regional health networks, home and community care organisations, on-call physicians networks, the regional telephone hotline for on-call physicians, an association for patient lung care, electricity distribution network operators, the regional general directorate of energy, the regional civil protection organisation, a city industrial services, the regional crisis management department, an organisation for electricity supply during crises, and telecommunication associations.

**FIGURE 2:**
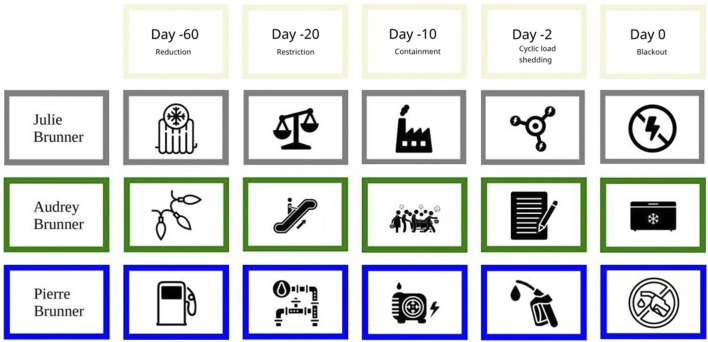
Cards illustrating the 5 scenes of the electricity shortage and blackout scenario involving three fictional characters working in different sectors of the society, used to accompany the narrative during the structured interview (Perceptions and Needs of Primary Healthcare Providers Regarding Electricity Shortages and Blackouts: A Qualitative Study Using a Realistic Narrative Approach, Switzerland, 2025). (“Power Generator” icon by Maurizio Fusillo, from thenounproject.com CC BY 3.0; “Radiator cold” icon by Smashicons, from thenounproject.com CC BY 3.0; “No Fuel” icon by Supanut Piyakanont, from thenounproject.com CC BY 3.0; “balance” icon by Alejandro, from thenounproject.com CC BY 3.0; “Escalator” icon by Krisna Arga Muria, from thenounproject.com CC BY 3.0; “fuel nozzle” icon by ProSymbols, from thenounproject.com CC BY 3.0; “pipeline” icon by Eucalyp, from thenounproject.com CC BY 3.0; “Shopping customers” icon by Gan Khoon Lay, from thenounproject.com CC BY 3.0; “no electricity” icon by Adrien Coquet, from thenounproject.com CC BY 3.0; “Industry” icon by Adrien Coquet, from thenounproject.com CC BY 3.0; “energy hubs” icon by Danku Sobieraj, from thenounproject.com CC BY 3.0; “Christmas” icon by Clea Doltz, from thenounproject.com CC BY 3.0; “paper and pencil” icon by Untashable, from thenounproject.com CC BY 3.0; “freezer” icon by Rafael Farias Leão, from thenounproject.com CC BY 3.0).

The information used to develop the ESI scenario are summarized in Table 1 and narratives used for each character in Table 2. Paper cards were used to support the scenario during health professionals’ interviews (Figure 2).

**TABLE 1:** Information retrieved from 16 key professionals who are part of preparation plans or would be involved in an electricity supply interruption (Perceptions and Needs of Primary Healthcare Providers Regarding Electricity Shortages and Blackouts: A Qualitative Study Using a Realistic Narrative Approach, Switzerland, 2025).

**Awareness of risk and levels of concern**
Almost all professionals acknowledged the reality of future electricity shortages or blackouts, though they varied in how imminent they believed the risk to be. An electricity distributor member explained how they train regularly for a network restart, even if they consider the odds low. A member of a regional association for home care mentioned a high level of preparation with an ambitious strategy to manage the risk of ES. Nevertheless, the sense of urgency fluctuates. One representative of the regional health department mentioned that after the period of high risk of the winter 2022/23, the motivation to continue working on electricity shortage preparation plans has overall decreased, due to a drop in attention once the immediate threat receded, even though the underlying risk persists
**Existing plans and frameworks**
All participants highlighted that multiple initiatives in the region are ongoing. The plan by the organization for electricity supply in crisis envisions progressive measures (conservation, usage restrictions, and contingent consumption) (Figure 1). The regional health department member mentioned that protocols and checklists had been sent to several groups of professionals, and that priority healthcare sites for electricity backup had been designated. The civil protection organisation emphasized the role of emergency meeting points (called in French points de Rencontre d’Urgence: PRU), who are each aimed to cover the needs of 5,000 habitants and are equipped with power supply and radio network to maintain a “discussion channel” if conventional phone services fail. However, coordination remains partial according to stakeholders
**Anticipated adaptations and foreseen challenges**
A regional electricity distributor representative highlighted that distribution lines were not expected to sustain the on and off switch of entire sections of the grid. Indeed, devices such as alarm systems, home automation systems, and antennas are prone to structural damage, since they are designed to operate under a constant voltage without any power interruption. As a result, they may fail to restart correctly, reset themselves, lose synchronization, or fail to power back on at all, thereby requiring human intervention. A member of the regional telephone hotline for on-call physicians mentioned that less calls are expected than usual during an ES or a black-out (as it happened during the COVID-19 crisis). Their plan is to manage non-vital healthcare demands via concise radio exchanges with the emergency meeting point (PRU). The latter would use 5 pre-defined questions allowing them to quickly assess the medical situation and direct patients to the closest and appropriate healthcare provider. The association for patient lung care has identified 500 patients critically dependent on electricity with some that might need relocation if the electricity outage is prolonged. Some home care organisations bought starlink internet antennas for healthcare providers to be able to gather key information from the facility management system at a pre-defined meeting point. A regional electricity distributor member underscored that segmenting the electrical grid for rotating load shedding could disrupt transport and communication flows, extending the impact of interruptions

**TABLE 2:** Narratives used for each character in the electricity shortage and blackout scenario (Perceptions and Needs of Primary Healthcare Providers Regarding Electricity Shortages and Blackouts: A Qualitative Study Using a Realistic Narrative Approach, Switzerland, 2025).

Day^a^	Character	Narrative
J-60	Julie	Julie is working as an engineer at the regional electricity control center. Tough winter is approaching. The swiss confederation decides that electricity saving is now essential. First, it encourages households to voluntarily reduce electricity consumption, for instance by lowering home temperatures, washing hands in cold water, or limiting the use of energy-intensive appliances like dishwashers and washing machines
​	Audrey	Audrey works for a supermarket and the store has made an important decision: shop windows will be turned off between 10 p.m. and 7 a.m. all year long. There’s no reason only households should make efforts. The supermarket is also repeating some previous actions, such as not decorating its facades during the christmas holidays
​	Pierre	Pierre manages a gas station and the swiss Confederation’s announcement doesn’t change much for him. Traffic remains heavy, even increasing
J-20	Julie	Weeks later, voluntary savings are insufficient. The Federal council issues ordinances to restrict non-essential energy-intensive uses. From her control center in Prilly, Julie works daily to balance electricity production and consumption. Events like festivals and ski lifts are now banned
​	Audrey	Audrey’s job has changed. Staffing has increased to handle new tasks: Without camera detection, she must check empty shelves manually and update prices using paper labels due to inactive electronic tags
​	Pierre	Pierre is reassured: gas stations are not among the restricted usages. Still, he wonders whether these early constraints will impact refinery production upstream
J-10	Julie	As winter tightens, Julie sees savings efforts yield limited results. The energy-intensive companies she monitors are now required to drastically reduce consumption, especially those using over 100 MWh per year
​	Audrey	Audrey learns that most of her coworkers are laid off. Only 1 in 5 supermarket stores will stay open, but unfortunately, not hers. Yet, her store remains crowded
​	Pierre	His station is still operational, but daily mobility has declined. Still, institutions contact him to procure diesel for emergency generators
J-2	Julie	January 17, 2024. Rotating outages begin due to a cold wave across Europe. Vaud is divided into 3 zones: each zone will be deprived of power for 4 h alternately, then re-powered for 8 h
​	Audrey	Today, Audrey’s Migros is powered from 8 a.m. to noon. She must stay at the store without knowing when systems will reboot. She’s injured but can't reach her doctor. Phone lines are down, and clinics are overwhelmed
​	Pierre	At 7:56 a.m., his station is powered for only 4 min, dozens of cars are waiting. Payment terminals are down. He’s forced to switch to manual payment and feels it’s unsustainable
J-0	Julie	An alarm goes off: the grid has collapsed. After days of instability, Julie and her team are exhausted and Vaud faces its first full blackout
​	Audrey	Despite the panic, Audrey returns to the store and joins five colleagues transferring fresh goods into backup-powered refrigerators. Diesel reserves may only last until the next day. Julie is afraid that the lack of food will lead to violence in the store
​	Pierre	Pierre is overwhelmed. Every day he turns away people asking for fuel. His reserves are low, and without power, fuel pumps and electronic systems are inoperable—the fuel is there but unreachable underground

^a^ Number of days before the occurrence of the blackout.

### Health Professionals’ Perceptions and Needs

Eight semi-structured interviews were conducted with primary healthcare providers at their workplace or home, allowing to approach data saturation in relation with the objective of the study (Table 3). Four broad themes emerged from their discourses: 1) perception of the plausibility of ESI happening in Switzerland and emotions related to these potential events; 2) willingness to be integrated into crisis plans, and adaptative strategies; 3) perception of the electricity dependence of their practice and during clinical management; and 4) needs to continue managing patients despite ESI.

**TABLE 3:** Characteristics of primary healthcare providers interviewed (Perceptions and Needs of Primary Healthcare Providers Regarding Electricity Shortages and Blackouts: A Qualitative Study Using a Realistic Narrative Approach, Switzerland, 2025).

Characteristics	Private practitioners^a^	Home-visiting nurses	Total
Male	2	1	3
Female	3	2	5
Experience <10 years	0	3	3
Experience >10 years	5	0	5
Age <40 years	0	1	1
Age >40 years	5	2	7

^a^ Including 3 general internal medicine physicians, one paediatrician and one nurse practitioner in obstetrics.

#### Perception of the Plausibility of ESI and Emotional Responses

Participants considered the initial phases of the ESI scenario (supply tensions and shortages) plausible. However, the final phases (cyclical load shedding and blackout) were perceived as unlikely, if not unrealistic. These perceptions were justified in various ways, ranging from the Swiss state’s inability to fail on energy issues to the inability to force the economic sector to reduce its electricity consumption. *“I imagine it could happen, but it’s hard to think about it. The later phases seem less likely to me. We don’t really want this part up to now. (pointing to the scenario cards for phases 3 and 4)”* (PP 1, F, age >40 y, experience >10 y).

Reactions to ESI situations varied widely between participants, from anxiety to confidence. Some felt overwhelmed by the idea of practicing without electronic patient records, while others saw potential solutions and even viewed the situation as an opportunity for creative problem-solving: *“For me, it all just creates anxiety. Anxiety and stress.”* (HVN 3, F, age >40 y, experience <10 y). *“I see it as an opportunity to learn and adapt. It’s a challenging situation; it can also be a learning experience.”* (PP 2, M, age >40 y, experience >10 y). Interestingly, most of the participants who felt rather confident had a previous experience of working in a low-income country, where they had to practice in a degraded operating mode: *“I lived in countries where we often had small power cuts. So, life doesn’t function at the same pace, but you get used to it, you cope with it, and you adapt.”* (PP 4, M, age >40 y, experience >10 y).

While some participants trusted in organized responses by the authorities and citizens, others envision profound social stress that could precipitate conflicts much more serious than those presented in the scenario. *“[…] people become nasty even before things get that far (pointing to the scenario’s reference to altercations in a supermarket). […] At some point, it’s war. That’s just how it is. If you have a dog that’s hungry and another dog with food, it will attack, even if they grew up together. We’re no different.”* (PP 3, F, age >40 y, experience >10 y).

#### Willingness to Integrate Crisis Management Systems and Adaptative Strategies

Many participants emphasised their commitment to maintaining patient care despite the lack of electricity. Overall, the interviews revealed a strong sense of solidarity, with primary healthcare providers showing a willingness to make themselves available to provide care in one way or another. They believed in their capacity to adapt, even during a blackout, highlighting the need to prioritize urgent cases while finding makeshift solutions for non-urgent appointments. *”I think I would come in anyway, just to welcome patients who show up at the door and let them know we’ll postpone their consultation until later if it isn’t urgent.”* (PP 4, M, age >40 y, experience >10 y). *“I would prioritize essential services and use alternative power sources if available. Communication with patients and colleagues would be crucial.”* (PP 5, F, age >40 y, experience >10 y).

Some participants emphasized the benefit to stay in their usual medical practice, where they have all their medical devices. Because patients know where they are located, the place itself could represent a stable refuge, while moving to a new consultation site could add more stress: *“We can consult anywhere, but if we’re just a hundred meters from the shelter, it makes more sense to tell them to come to the practice, where we have the instruments.”* (PP 5, F, age >40 y, experience >10 y). Others mentioned their agreement to be rather integrated into electricity-powered medical or non-medical infrastructures, provided they would be informed in advance, and these places adapted to crises’ situations*. “If it's organised, then yes, why not? But it would need to be organised a little bit in advance (laughs). You can't improvise that on the day there's a blackout, that's for sure.”* (PP 4, age >40 y, experience >10 y). There was also a desire to maintain home visits. *“If someone in the community needs help or if there’s a situation nearby and I still have enough fuel, I can travel a bit further. I’d make myself available to help people at home or at least evaluate them if they’ve had a fall or a respiratory problem or anything like that.”* (HVN 2, M, age >40 y, experience >10 y). *“Maybe I’d increase my visits, just to reassure them? […] Those would be safety visits. Sometimes we do safety visits just to check if the person is okay.”* (HVN 1, F, age <40y, experience <10y). Adaptive behaviours would continue beyond the ESI period: medical practices could remain disrupted for several days thereafter, for example due to the necessity to integrate medical and administrative patient data recorded on paper during the crisis. *“If the blackout goes on for a while, we’ll still need to figure out how to keep track of our files, and also billing, because we won’t be able to catch up on everything if it lasts weeks or months.”* (PP 5, F, age >40 y, experience >10 y).

#### Perception of Electricity Dependency in Their Daily Practice

Most participants realized their practice relied heavily on electrical, digital and online services. As most data related to invoices, lab tests, prescriptions, appointment scheduling, patient files, and equipment orders circulate in a computerized format, a system-wide failure of information exchange was perceived as critical. *“[…] everything we have is computerized. Everything about patient follow-up is electronic. So, if we lose that, it becomes really complicated. We’d have to pretend we don’t know our own patients.”* (PP 1, F, age >40 y, experience >10 y). However, the consequences of a lack of access to electronic medical records varied by type of health problem: past history was perceived as much more essential for chronic than acute cases. *“So if it’s an emergency, we absolutely don’t need to have access to all the patient’s information. We don’t absolutely need our IT system. Because when it’s an emergency, it’s a very specific problem, affecting a particular organ, and we can manage without necessarily knowing every detail about the patient […] It’s more for chronic situations, where you have a file with 25 pre-diagnoses that you have to take into account when deciding which investigations to do […] It’s more in that kind of situation that a lack or shortage of electricity starts to become problematic.”* (PP 4, M, age >40 y, experience >10 y).

For nurses making home visits, they could easily take patient follow-up notes by handwriting, while the need for real-time phone or text messages exchanges with in-charge physicians, other colleagues or headquarters to know the visits plan was considered essential. *“No, well, transmissions between nurses, we do them by phone. We don’t share our written materials. […] And if my colleague shows up on site and there’s some information she’d have liked to know and that’s nagging at her, she just calls me, sends a message on WhatsApp”* (HVN 1, F, age <40 y, experience <10 y). Permanent access to a functional communication network was thus considered crucial. *“No, I often communicate with doctors by email. I send emails giving them an update on the situation. I rarely speak to doctors on the phone. I know that some of my colleagues call more often, but I rarely do. I also often have contact with physical therapists and occupational therapists.”* (HVN 3, F, age >40 y, experience <10 y). If this would not be possible, home-visiting nurses highlighted the legal problem of not being authorized to perform medical procedures without confirmation by a physician. *“[…] the doctor needs to be aware of what is happening at home. For example, if there is a wound, I cannot change the dressing with my own equipment without informing the doctor and without complying with and following medical protocol. I cannot just do whatever I want and ignore the doctor. We have to work together.”* (HVN 1, F, age <40 y, experience <10 y).

Professionals realised during the interview that almost all their medical devices were electricity-dependant, including thermometers, weighing scales or blood pressure monitors. It was however not a major concern, even regarding lab tests. *“I could manage quite well for a few weeks without the lab.”* (PP 5, F, age >40 y, experience > 10 y). Some participants highlighted their ignorance of which practices are energy-intensive and how to contribute to energy saving. *“Afterwards, ideally it would be to have perhaps… a base to know what requires a lot of energy. To know what we could focus on to participate in this energy saving. Because I’m a nurse, I don't realize what uses more power than anything else. I don’t realize it.”* (HVN 1, F, age <40 y, experience <10 y). Some participants had already developed personal strategies for resilience regarding their equipment, stemming from a distrust of electronic devices or desire to maintain independence from digital systems (e.g., proactively keeping paper-based medical records or medical books). *“I have an electric device, but I always have my manual device with me because my electric device, 1 day it will run out of batteries. And I don't necessarily have batteries with me. Plus, I have a bit of trouble trusting electric devices.*” (HVN 3, F, age >40 y, experience <10 y). *“We are happy to still be on paper files (laughs). Because there’s so much pressure to switch to an electronic file. And we stick to our paper files precisely for that [to reduce our dependence on IT].”* (PP 2, M, age >40 y, experience >10 y).

Several people raised the issue of lighting. Some suggested solutions ranging from reorganising the working day to installing additional lighting: “*I’d probably set up some candles to have a little light.”* (HVN 1, F, age <40 y, experience <10 y*); “But that’s exactly when we realized that if we shifted our schedule just a little bit—starting just a bit later—we’d have plenty of daylight”* (PP 2, M, age >40 y, experience >10 y). When it comes to telecommunications, adapting seems more difficult and expectations of authorities’ interventions are high: *“I’m waiting for them [the cantonal level] to handle this kind of thing in terms of communication; there will still be some issues to deal with, etc.”* (PP 5, F, age >40 y, experience >10 y); *“Anyway, I couldn't ask anyone since communications are down. So we’re in a situation where everyone will just have to fend for themselves.”* (HVN 2, M, age >40 y, experience >10 y). Regarding the cold chain, research into vaccines that can withstand a rise in temperature is being proposed: *“With the proper refrigerators, that means they can guarantee that the vaccines will remain cold for a certain number of hours. And now we’re also looking for standard vaccines that can be stored at room temperature for longer periods, such as Vaxelis, which can remain outside the refrigerator for 150 h”* (PP 2, M, age >40 y, experience >10 y).

#### Needs Identified by Participants to Cope With ESI

Most professionals were unaware of plans such as INOPIA, the PRUs or the OSTRAL plan. Despite an official email sent a few months before by local health authorities to physicians and home care organisations regarding potential electricity shortages (that included checklists), most participants could not recall receiving this information. They had therefore a feeling of a lack of communication between authorities and professionals in the field. *“I had never heard of any protocols before. I didn't know about these protocols.”* (HVN 1, F, age <40 y, experience <10 y). When showing them the checklists, some participants reacted positively, finding such tools useful and highlighting possible adaptations. *“If there are organized procedures, I would be willing to follow them and adapt my practice accordingly.”* (PP 4, M, age >40 y, experience >10 y). However, concerns were raised about their practicality, especially regarding printing consultations summaries for all patients, which was deemed impossible and wasteful. There was also apprehension about the burden this would place on medical assistants who would have already many other additional tasks in case of ESI. *“It’s impossible because we all have 1000 patients, so if we have to print them times five, imagine!* [practice with 5 physicians] *And then, all those who won't come during this period, it’s really a waste. And then we ask our assistants to print and that’s it, we have five resignations”* (PP 1, F, age >40 y, experience >10 y).

Only one participant - a member of municipal council - was familiar with the concept of PRUs and understood their potential role. Regarding their capacity in playing a first aid role, she commented: *“I don't think my patients would go to the meeting points; they would prefer to stay at home. Without electricity, they would wait… Perhaps I would increase home visits to reassure myself.”* (HVN 3, age <40 y, experience <10 y).

Participants were unaware of crisis plans involving private practices. *“I don't know if such plans are being discussed, but in any case, for the moment,* I've *never heard of an emergency plan where practising doctors would be recruited to work in emergency rooms in hospitals, for example. No, I haven't heard anything about that.”* (PP 4, age >40 y, experience >10 y). Several envisioned professional associations playing a networking and facilitating role. They suggested these associations should anticipate crises, communicate with relevant health partners, and facilitate information exchange during crises, offering a comprehensive view of the professional landscape necessary for quality care. They were however sceptical about their reactivity and ability to provide relevant help. *“Well, from the regional society of medicine, I expect them to communicate. But since they’re always late… I'm not sure they’d be of much help to us […] They didn’t communicate anything to us, it was ‘Oh, look with the national society of medicine.’ Anyway, the national society of medicine’s recommendations were impractical, unreadable. So we didn't use them at all.”* (PP 5, F, age >40 y, experience >10 y).

## Discussion

Primary healthcare providers expressed a strong willingness to maintain continuity of health services during a hypothetical electrical crisis. To be able to anticipate and face it, they however emphasized the importance of receiving information, assistance and realistic guidelines from public health actors. The interviews, along with the narrative approach based on an ESI scenario, seemed to act as catalysts for reflection, prompting participants to assess their own roles, vulnerabilities, and potential strategies. For many, this exercise seemed to help them translate a hypothetical situation into real-world sensitisation. These data support the existing evidence showing that narrative story can stimulate prospective thinking and contribute to anticipatory resilience planning [16].

Perceptions of the plausibility of ES and blackout varied. While early phases of the ES plan were deemed realistic, blackouts were met with scepticism. This optimism toward containment may reflect a broader confidence in Swiss infrastructure and governance. However, the belief that electricity will be consistently available may also lead to underestimation of systemic vulnerabilities. Their perceptions regarding populations’ potential reactions during crises (major conflicts and acts of violence) appear pessimistic and do not correspond to the literature based on observations from previous crises, where acts of solidarity were predominantly observed [17, 18].

A lack of knowledge and preparation were noticed, with participants not aware of existing crises strategies and protocols. Participants emphasized that communication with health authorities and other actors was a challenge. Fostering a culture of risk within healthcare providers, conducive to dialogue among all stakeholders and professionals is essential to achieve a realistic plan [19]. Participants also noticed that checklists provided by local authorities were rather disconnected from their practice. Thus, to consider and involve healthcare professionals to explore different communication pathways, determine the message to be conveyed and develop procedures and checklists is necessary.

Despite lack of knowledge and preparation, a strong willingness to integrate into structured responses emerged. Providers wanted realistic, actionable, and localized decisions and actions. They had a spontaneous preference to remain in their usual work environment, which was perceived as a secure and familiar space for both staff and patients. Some were however open to the idea of working in adapted infrastructures, provided they would be informed in advance. This supports the idea of reinforcing decentralized, resilient models of care delivery [20, 21], not only to improve health systems in general but also to face crises and become more resilient towards systemic risks [as observed during the COVID-19 pandemic [22]]. Interestingly, none of the interviewees mentioned the option of simply closing their practice, to be able for example, to take care of their family at home. This important dilemma faced by professionals during crises is obviously a very sensitive topic that might have been difficult to raise spontaneously.

Our study confirms the strong dependence on electricity: while many primary healthcare providers felt confident managing acute care without access to electronic medical records, they anticipated that the loss of digital tools might significantly impair chronic care management and coordination. Digital fragility also emerged through the centrality of IT platforms, email, cloud-based medical records, and online billing. The digital needs of physicians’ practices were different from that of nurses making home visits, the latter facing the legal problem of not being allowed to undertake medical procedures without physician’s confirmation. A blackout could disconnect healthcare teams not only from their patients but also from each other. Future preparedness should consider energy-resilient alternatives like keeping paper-based key patients’ medical information, local IT data storage, hard-copy protocols, or low-tech backups [23]. Some participants appear to anticipate certain indirect effects that a blackout might cause, as well as practical solutions to address them: the lack of lighting or the need to keep certain medications refrigerated. However, some indirect effects were not reported, such as the failure of electric pumps, which would bring the drinking water network to a standstill. This would seriously compromise patient hygiene, thereby increasing the risk of disease, and certain care practices would become impossible [4, 8].

Few participants spontaneously mentioned structural strategies to reduce electricity dependency, an issue highlighted in energy resilience research [24]. This gap suggests the need to broaden the preparedness conversation from individual responses to system-level changes. The use of scenario narratives could be useful tools for such programs. These scenarios could explore different causes of ESI, including climate related extreme weather events such as those experienced by mountain regions in Switzerland recently. Even if, for healthcare providers and centres, the origin of the shortage does not change fundamentally the consequences on their practice, the geographical extent of the affected area will still modify the extent of the impact (the latter being less in the case of a localized interruption due to a climate related disaster compared to nation-wide blackout due to imbalance between supply and demand).

### Limitations

We interviewed only a limited number of primary healthcare providers. Findings may therefore not be generalizable to all primary healthcare settings or professionals. Diverse gender, age and professional experience were represented in our study but relationships between sociological characteristics of participants and their perceptions were not analysed. Finally, because participants were not aware of existing checklists and discovered them during the interviews, they had no time to read them in detail.

### Conclusion

This study highlights the need for structural changes to allow a better communication and cooperation between public health actors and primary healthcare providers in the building of realistic crisis management strategies. Training and preparation sessions specifically designed for primary care are needed, along with plans explaining where each healthcare provider could best contribute to the continuity of primary care, which is essential to prevent hospitals from becoming overwhelmed. The scenario crafted in this study could be enhanced to raise awareness of the many consequences and adaptation possibilities in the event of a blackout. Redefining the role of primary healthcare providers in crises represents also a key opportunity to enhance the resilience of the whole healthcare sector.
